# Regulation of Innate Immune Signaling by Autophagy

**DOI:** 10.3390/ijms27125413

**Published:** 2026-06-16

**Authors:** Daniel Oña-Sánchez, Julia Bandera-Linero, Felipe X. Pimentel-Muiños

**Affiliations:** Centro de Biología Molecular Severo Ochoa (CBM, CSIC-UAM), Consejo Superior de Investigaciones Científicas, Universidad Autónoma de Madrid, Nicolás Cabrera, 1, 28049 Madrid, Spain

**Keywords:** innate immunity, pathogen-associated molecular patterns (PAMPs), inflammation, pattern recognition receptors (PRRs), selective autophagy, danger associated molecular patterns (DAMPs), protein degradation

## Abstract

The first line of defense against infection is provided by the innate immune system, which is able to recognize molecular patterns in a variety of infectious agents through the action of different families of pattern recognition receptors (PRRs). These effectors detect the invading agent and trigger powerful inflammatory responses that help fight the infection from the very beginning. However, inflammatory reactions can be damaging for the host and must be properly controlled to prevent pathological consequences. Here we provide a comprehensive review of the important role of autophagy, a catabolic pathway that degrades cellular components for quality control and regulatory purposes, in the regulation of innate immune responses, and the underlying mechanisms involved. Inflammatory pathways discussed in this review include those triggered by Toll-like receptors (TLRs), Retinoic acid-Inducible Gene (RIG)-I-like receptors (RLRs), Nucleotide-binding Oligomerization Domain (NOD)-like receptors (NLRs), and the receptor for cyclic GMP–AMP Stimulator of Interferon Genes (STING). Finally, we also consider examples where autophagy plays context-dependent or even pro-inflammatory roles, reflecting a complex involvement that remains to be fully characterized.

## 1. Introduction

Innate immunity constitutes the first line of defense against infectious agents. This early response is mediated by a diverse array of receptors that trigger inflammatory responses upon recognition of common molecular patterns present in potentially pathogenic microorganisms (called pathogen-associated molecular patterns, PAMPs) [1]. These receptors can also recognize endogenous molecules (named danger-associated molecular patterns, DAMPs) that become exposed upon cellular injury or stress to trigger inflammation as a sign of alarm [2]. Families of such pattern recognition receptors (PRRs) include the transmembrane Toll-like receptors (TLRs), the cytoplasmic sensors Retinoic acid-Inducible Gene (RIG)-I-like receptors (RLRs) and Nucleotide-binding Oligomerization Domain (NOD)-like receptors (NLRs), and the DNA detector cyclic GMP–AMP synthase (cGAS) that operates through Stimulator of Interferon Genes (STING) [3]. Signaling induced by PRRs triggers pro-inflammatory and immunoregulatory pathways that not only provide a first defensive response but also set the stage for the onset of adaptive immunity by delivering helper signals for T cell differentiation and maturation [4]. The potent pro-inflammatory activity of PRRs needs to be tightly controlled to prevent inflammatory pathology, as dysfunction of negative regulatory mechanisms leads to pathological phenotypes in a variety of experimental systems and also in patients [5].

Autophagy is a catabolic pathway that eukaryotic cells utilize to degrade their own components in a regulated manner [6]. This process involves the formation of prototypical double-membrane vesicles (called autophagosomes) that enclose cytoplasmic cargo and eventually fuse with lysosomes to degrade their contents. The core molecular machinery that regulates autophagy in mammalian cells has been well characterized and includes a number of autophagy-related (ATG) proteins that act in concert to induce autophagosome formation [7]. The conventional pathway that promotes autophagy in response to nutritional stress is initiated by inhibition of mechanistic target of ramapaycin (mTOR), a protein kinase that regulates cell growth. Such inhibition induces the activation of two protein complexes (ATG13-ATG1-FIP200 and ATG6-ATG14-VPS34) that initiate autophagosome nucleation. The expansion and final closure of the autophagosomal sac is promoted by two ubiquitin-like modification systems that converge in a final ATG16L1-5-12 complex which, in turn, drives the lipidation of ATG8/LC3 to generate a membrane-bound form called LC3-II. The presence of LC3-II in the autophagosomal membrane is important for completion of the autophagosome and its maturation for fusion with the lysosomal compartment [8]. Due to important roles in stress management and quality control, autophagy is widely viewed as a mechanism that helps maintain healthy cellular homeostasis [9]. In addition to this canonical pathway, a number of unconventional autophagic processes induced by alternative uses of the canonical machinery and/or serving non-degradative functions have also been described, often involving the labeling of single membrane compartments with LC3 without the intervention of the upstream protein complexes that initiate autophagosome formation [10]. These mechanisms mediate a variety of cellular functions like unconventional protein secretion, restoration of lysosomal dysfunction, the control of endosomal trafficking (LC3-associated endocytosis, LANDO) or the targeting of phagosomes to the lysosomal compartment (LC3-associated phagocytosis, LAP) (reviewed in [11]).

Autophagic degradation of random cytoplasm is a common response to nutritional stress, and in this physiological context the main purpose of autophagy is to redirect resources from superfluous cellular components to more critical processes. Thus, in this case, the basic components that result from the degradation process are recycled to sustain macromolecule synthesis or important metabolic pathways. On the other hand, selective forms of autophagy promote the specific elimination of obsolete or potentially damaging cellular components, like depolarized mitochondria, invading microorganisms or toxic protein precipitates [12]. In many of these cases, the specific substrates become ubiquitinated and adaptor proteins containing both ubiquitin-binding domains and LC3-interacting regions (LIRs), like p62/sequestosome-1 (SQSTM1), neighbor of BRCA1 (NBR1), TAX1BP1, nuclear dot protein 52 (NDP52) or optineurin (OPTN), target them to incipient autophagosomes for sequestration and subsequent degradation [13]. However, selective autophagy also targets specific proteins for destruction to control a variety of cellular processes [14]. In this case, the underlying mechanisms also frequently involve substrate ubiquitination, but some of them are mediated by direct interaction between the substrates and selective autophagy receptors or LC3.

Substantial evidence indicates that absence of autophagy causes increased inflammation in different of experimental systems, both in vitro and in vivo [15,16]. For example, early studies showed that deletion of *Atg16l1* enhances TLR signaling and susceptibility to intestinal inflammation [17], and depletion of ATG5, ATG7 or ATG16L1 in the intestinal epithelium causes an inflammatory pathology that is reminiscent of human Crohn’s disease [18]. One important underlying mechanism through which autophagy restrains inflammation is linked to a general role in the elimination of stimuli that trigger innate immune pathway activation, like invading bacteria (xenophagy), damaged mitochondria (mitophagy) or viral replication intermediates. In addition, it is becoming clear that autophagy also dampens inflammatory signaling by selectively degrading key signal transduction effectors in order to keep the pro-inflammatory pathways in check. Innate immune signaling pathways regulated by autophagy include those triggered by TLRs, NODs, RLRs and cGAS/STING PRRs. In the case of intestinal inflammatory conditions like Crohn’s disease, multiple roles of autophagy in different cell types appear to act coordinately to suppress pathological inflammation. These roles include, as mentioned above, the elimination of invading microorganisms by intestinal epithelial cells via xenophagy and the control of innate immune signaling in the myeloid compartment, and sustaining the secretion of mucus and anti-microbial peptides by Paneth and Goblet cells, which aid in the defense of the intestinal epithelium against potentially pathogenic members of the intestinal flora [19].

Here we provide a comprehensive and up-to-date revision of the role of autophagy as a mechanism that operates at multiple levels to control inflammatory reactions, including the removal of initiators of innate immune signaling as well as examples of the specific degradation of different PRR signaling mediators. We also discuss a few paradoxical examples where autophagy appears to have pro-inflammatory consequences, thus underscoring the complexity and context-dependent functions of the autophagic pathway in this context.

## 2. Role of Autophagy in the Removal of Innate Immune Stimuli

**Figure 1:**
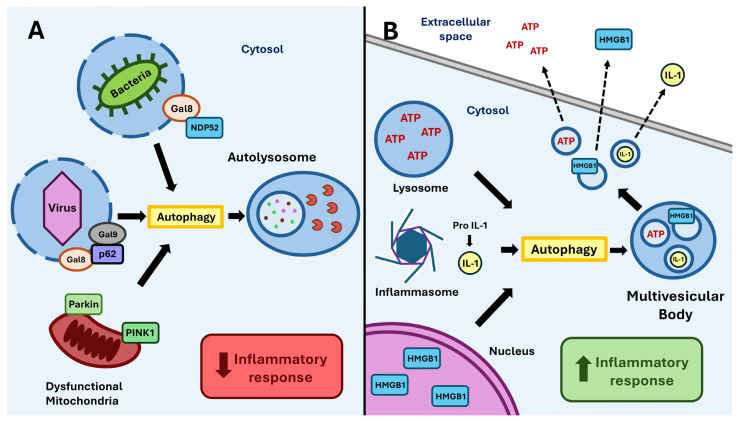
Dual role of autophagy in controlling innate immune response. (**A**) Selective autophagy limits immune responses by targeting inflammatory stimuli for lysosomal degradation. Autophagy receptors such as p62 and NDP52, together with vesicle damage sensors of the galectin family, recognize invading bacteria and viruses and deliver them to autophagosomes for subsequent lysosomal clearance. Damaged or dysfunctional mitochondria are selectively eliminated via PINK1–Parkin-mediated mitophagy, reducing their capacity to drive inflammatory responses. (**B**) Autophagy regulates the secretion of inflammatory mediators. Under apoptotic stress, both HMGB1 and ATP (DAMPs liberated during apoptotic cell death) are redistributed from the nucleus and lysosomes to autophagosomes and MVBs, respectively, for extracellular release in an autophagy-dependent manner. Similarly, the autophagic machinery regulates mature IL-1b secretion by promoting its trafficking to MVBs and subsequent extracellular liberation.

Innate immune receptors detect the presence of PAMPs during bacterial or viral infection but are also stimulated by endogenous DAMPs, and many examples indicate that specific forms of autophagy mediated by selective autophagy receptors play an important role in eliminating such stimuli and their sources to prevent inflammatory responses (Figure 1A). Substrate specificity is generally provided in this context by the individual mechanisms that induce decoration of the specific cargo with poly-ubiquitin chains or LC3-interacting proteins, guaranteeing selective targeting to incipient autophagosomes [20]. While this is not the main focus of this review, we provide a brief categorization and discussion of these mechanisms. The presence of intracellular bacteria, whether free in the cytoplasm or inside bacterial vesicles, is a major source of innate immune activators, including cell wall components (lipopolysaccharide, LPS; muramyl dipeptide, MDP), flagellin or bacterial DNA [3]. Selective forms of autophagy target invading bacteria for degradation (a process which is generically called xenophagy) through a variety of mechanisms [21,22]. For example, bacterial phagosomes can be engulfed by canonical autophagosomes as if they were regular cargo to produce multimembrane autophagic compartments that degrade the intruder [23]. Single-membrane phagosomes are directly conjugated with the autophagosome marker LC3 via an unconventional mechanism called LC3-associated phagocytosis (LAP), a phenomenon that promotes their fusion with the lysosomal compartment (reviewed in [24]). Some invading bacteria rupture their phagosome to actively replicate in the cytoplasm. In these cases, they become rapidly ubiquitinated for recognition by selective autophagy receptors like p62/SQSTM or OPTN and subsequent engulfment by canonical autophagosomes [22]. In addition, ruptured phagosomes or bacterial vesicles are detected by galectins (particularly galectin-8), which are lectins able to recognize the complex glycans that are only present in the outer side of the plasma membrane or in the lumen of vesicular compartments [25]. Once damaged bacterial vesicles are detected, galectins bind the selective autophagy adaptor NDP52 to deliver both the damaged vesicle and the bacteria inside for autophagic degradation. Importantly, this surveillance system also operates for sterile vesicular damage, so it constitutes a broad mechanism able to detect the presence of both PAMPs and DAMPs that compromise endosomal and lysosomal integrity.

Viral infection is detected by several PRRs. Thus, specific TLR and RLR family members react to viral RNA replication intermediates or surface glycoproteins, and the cGAS/STING signaling system is activated in response to cytoplasmic viral DNA [26]. Selective autophagic degradation of viral components helps dampen viral replication and therefore diminishes PRR activation and inflammatory signaling during the infection process. For example, galectin-9 promotes degradation of hepatitis B virus core proteins through a mechanism dependent on p62 [27], and galectin-8 targets porated endosomes containing picornaviruses RNA for degradation [28]. A p62-dependent pathway clears the capsid protein of Sindbis virus [29] with the intervention of SMURF1, an E3-ubiquitin ligase that ubiquitinates the capsid protein and targets it to p62-positive autophagosomes to control the infective process [30]. Similarly, TLR7- and RIG-I/Mitochondrial antiviral signaling protein (MAVS)-induced autophagy reduces the replication of the Rift Valley Fever virus [31], and SCOTIN, an interferon-induced protein that is expressed in the endoplasmic reticulum, restricts Hepatitis C virus replication by interacting with the essential factor NS5A and delivering it for autophagic destruction, behaving in this context as a selective autophagy receptor [32]. Finally, Fanconi Anemia proteins have a surprising role in promoting the degradation of capsid proteins of the Sindbis and Herpes Simplex viruses, acting as selective adaptors to promote their targeting to autophagic vesicles [33]. In addition to inducing autophagy during the infective process through PRR activation, viral infection can also activate autophagy mediated by a direct interaction between SNX5 and the Beclin/ATG14/PI3KC3 complex that increases its autophagosome formation activity [34]. These data, considered together, suggest that induction of autophagy could constitute an effective antiviral therapeutic strategy. However, additional evidence indicates that viruses have developed their own tools to promote the autophagic degradation of innate immune effectors as a way to resist their anti-viral activity. For example, influenza and pseudorabies viruses encode a protein called PB1 that facilitates MAVS degradation by recruiting NBR1 or NDP52, respectively [35,36]. Therefore, autophagy can play a double-edged sword in the biology of viral infections, and its consideration as a valid therapeutic strategy is not straightforward and should be evaluated in a context-dependent basis.

Damaged mitochondria are a source of mitochondrial DNA (mtDNA) and reactive oxygen intermediates (ROS), both of which are detected as DAMPs by various PRRs. Dysfunctional mitochondria are delivered for degradation by a specialized form of autophagy called mitophagy [37], thus helping minimize inflammatory signaling triggered by DAMP emission. Mechanistically, damaged mitochondria are recognized by the PINK/Parkin surveillance system and become ubiquitinated for interaction with selective autophagy receptors. PINK is actively imported and degraded by healthy mitochondria, but mitochondrial depolarization stabilizes it at the outer mitochondrial membrane where it phosphorylates and activates Parkin, an E3 ligase that causes extensive ubiquitination of mitochondrial proteins for autophagic targeting primarily mediated by NDP52 and OPTN [38]. Alternative mechanisms involve direct recognition of the damaged mitochondria by LIR-containing proteins like BNIP3, FUNDC1 or PHB2, which are specifically induced in response to different insults and bind LC3 at the surface of incipient autophagosomes for mitochondria sequestration and subsequent degradation. Finally, mitochondria-derived vesicles (MDVs) can also deliver portions of damaged mitochondria to the lysosomal compartment, although this route appears to proceed independently of the autophagic machinery [39]. Mitophagy efficiently inhibits the activation of the NLRP3 inflammasome by preventing the release of mtDNA through the induced degradation of the dysfunctional mitochondria [40,41]. At least in some cases, this function is mediated by p62 induced by the same NF-kB pro-inflammatory signaling that primed NLRP3 activity in the first place [42]. In some instances, unwanted cytoplasmic DNA can be selectively delivered to autophagosomes, although the mechanistic details remain elusive [43]. In addition, ROS generated by dysfunctional mitochondria activate NLRP3 to promote its translocation from the endoplasmic reticulum to perinuclear structures partially colocalizing with the damaged mitochondria, and in this scenario the active autophagic elimination of ROS-producing mitochondria helps prevent inflammasome activity [44,45,46].

## 3. Regulatory Role of Autophagy in the Release of Inflammatory Mediators

Autophagy has been involved in the mobilization of endogenous DAMPs to shape the immunogenic properties of programmed cell death (Figure 1B). For example, the liberation of the nuclear non-histone protein High mobility group box 1 (HMGB1), a DAMP released by apoptotic cells which is able to engage various TLRs, has been shown to rely on the autophagic machinery [47] for its trafficking to autophagosomes and multivesicular bodies (MVBs) and subsequent secretion [48]. Similarly, autophagy is required for ATP release during apoptosis to promote the immunogenic features of this cell death modality [49,50]. Mechanistically, ATP redistributes from lysosomes to autophagosomes to be secreted through a LAMP1- and Pannexin-dependent mechanism [51]. However, in contrast with this positive effect, unconventional forms of autophagy appear to prevent the liberation of ATP during apoptotic cell death by sequestering it into unconventional autophagic vesicles bound by just one membrane (and therefore different from canonical double-membrane autophagosomes) [52], indicating that the autophagic machinery regulates ATP liberation at different levels. Unconventional forms of autophagy are also required for optimal anti-inflammatory signaling provided by the cytokine IL-10 [53], thus contributing to a pathological inflammatory response in the event of dysfunction. Autophagy has also been involved in the release of the pro-inflammatory cytokine IL-1β from primary macrophages in response to inflammasome stimulation [54], or from highly transfectable HEK293 cells reconstituted with inflammasome components [55]. In the latter case, IL-1β is proposed to be internalized into cytoplasmic vesicles that later turn into double-membrane autophagosomes, leading to a proposed model where the cytokine becomes trapped in the intermembrane space that separates the two autophagosomal membrane layers. This structure would subsequently be trafficked for fusion with MVBs and final secretion [55]. In fact, autophagy has a broader role in the unconventional secretion of leaderless proteins, often involving cargo trafficking to autophagosomes and MVBs [56]. However, the role of autophagy in IL-1β liberation is more complex, since the autophagic pathway is also known to negatively regulate the levels of the unprocessed precursor by promoting its degradation in response to TLR signaling, thus limiting inflammation (Ref. [57]; see below). Therefore, the net effect of autophagy in IL-1β secretion likely depends on the biological context. In any case, more recent studies have shown that IL-1β is primarily released through Gasdermin D pores in both healthy and pyroptotic cells [58], and how this mechanism interacts with the roles of autophagy mentioned above remains to be fully elucidated [59]. In sum, the autophagic machinery regulates the mobilization of inflammatory mediators at multiple levels (Figure 1B).

## 4. Autophagic Control of TLR Signaling

TLRs are transmembrane receptors that recognize the presence of PAMPs in the extracellular medium or in intracellular vesicular compartments [60]. As mentioned above, early evidence of an important role of autophagy in the negative control of TLR signaling was provided by Saitoh et al. [17] in a seminal publication showing that absence of autophagy caused by deletion of *Atg16l1* results in increased inflammasome activation and IL-1/IL-18 release induced by endotoxin. The same authors described that this effect requires the elevated levels of ROS that are found in autophagy-deficient conditions, likely due to poor quality control of damaged mitochondria. These results are consistent with a model where autophagy eliminates faulty mitochondria to keep ROS levels to a minimum and prevent inflammasome activation and IL-1 processing for secretion after TLR stimulation.

**Figure 2:**
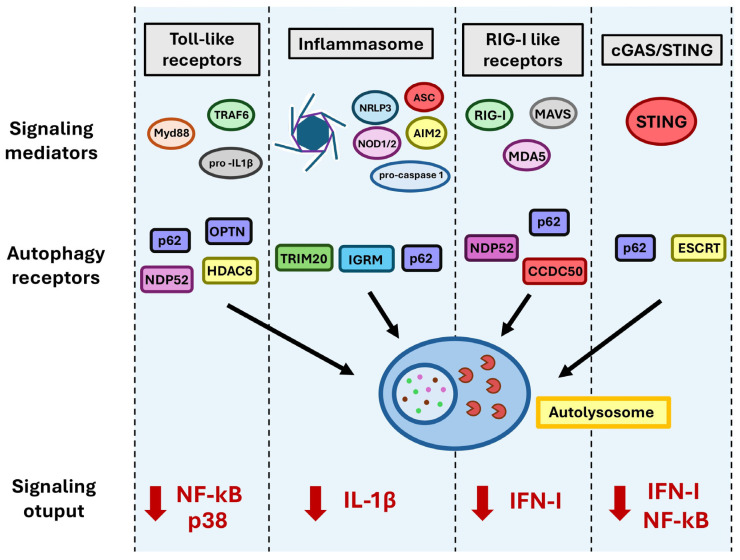
Negative regulation of innate immune signaling by autophagy. Selective autophagy negatively regulates innate immune signaling by targeting key pathway mediators for lysosomal degradation. Myd88, TRAF6 and pro-IL-1b are targeted for autophagic clearance downstream of Toll-like receptor activation by the selective receptors p62, NDP52 and OPTN, in concert with HDAC6. Inflammasome components and NOD-like receptors are eliminated through TRIM20, IRGM and p62-mediated selective autophagy. The activity of the MAVS/RIG RNA sensing pathway is controlled by autophagy through the direct degradation of RIG-I, MAVs and MDA5 mediated by p62, NDP52 and CCDC50 selective receptors. Finally, the DNA sensing pathway involving cGAS/STING is regulated by p62 and ESCRT-mediated autophagic degradation of STING.

Subsequent publications point to a relevant role of selective autophagy receptors in the degradation of TLR signaling mediators as a mechanism of negative regulation (Figure 2). Thus, SQSTM1/p62 and the histone deacetylase HDAC6 promote the aggregation of the TLR signaling effector MyD88 into large aggregates that suppress TLR signaling to p38 and JNK (though not substantially to NF-kB) and also delay TRAF6 recruitment to the MyD88 signaling complex [61]. Similarly, the TRAF6/TRIF complex becomes aggregated and degraded by autophagy through the selective autophagic receptor NDP52 in response to TLR3 activation by poly(I:C), thus suppressing NF-kB and IRF3 activation. Intriguingly, this effect of NDP52 is only observed in the absence of the inducible ubiquitin editor A20, suggesting that this control mechanism operates in particular physiological settings [62]. An additional selective autophagy adaptor (OPTN) has been described to bind IRAK1 to prevent the polyubiquitination of TRAF6, a mechanism that dampens NF-kB activation in response to LPS without involving a degradative activity [63]. Similarly, in the context of TNF signaling, OPTN also binds poly-ubiquitinated RIP to impede RIP1-NEMO interaction and activation of NF-kB [64], although the relevance of this mechanism in TLR signaling has not been confirmed. In addition, TLR-induced autophagy favors sequestration of pro-IL-1β inside newly generated autophagic vesicles where it becomes degraded, thus reducing the amount of IL-1β precursor available to the inflammasome for processing and subsequent release [57]. TLR signaling can also be downregulated through a direct interaction between Lamtor5 and activated TLR4 that targets both molecules to the autolysosomal surface. In this location, they prevent mTORC1 activation and favor an autophagic response that promotes TLR4 degradation, thus limiting downstream inflammatory responses [65]. In addition to these examples of negative regulation, autophagy has also been shown to promote TLR signaling by sequestering cytoplasmic PAMPs and delivering them to endosomal compartments for activation of those TLR family members naturally present in them [66,67].

Interestingly, a more recent publication has described that the signaling process induced by most TLRs involves the assembly of a large-scale signaling platform formed by aggregates of MyD88 (called myddosomes) [68]. This structure is assembled early in the process and provides a platform for the hierarchical and dynamic recruitment of the signal transduction mediators that participate in the pathway, including IRAK, TRAF6 and downstream kinases like MAPKs and IKK. The complex evolves in size and components over time and, intriguingly, in a late stage, it recruits autophagic effectors like ATG16L1, p62 or LC3 to mediate myddosome degradation and clearance. In this study, the authors found no influence of this final autophagic step on the overall signaling output, but it might have more functional significance in other systems. Of note, this scenario bears similarities with the STING pathway, where the late autophagic degradation of active STING suppresses signaling (see below).

## 5. Role of Autophagy in Inflammasome Regulation

Inflammasomes are large molecular complexes that typically include one family member of the cytoplasmic Nucleotide-binding Oligomerization (NOD)-like (NLR) group of PAMP sensors, an adaptor protein (ASC) containing CARD and PYD domains, and caspase-1 [69]. These complexes are assembled in the presence of PAMPs or DAMPs and promote caspase-mediated processing of previously induced IL-1 and IL-18 for secretion though gasdermin D pores, which are also formed by inflammasome activity [70]. Thus, the function of the inflammasome is subordinated to the action of a previous inflammatory stimulus (typically delivered by TLRs) that increases the expression of inflammasome components and immature IL-1β (pro-IL-1β), which cannot be secreted unless processed by the inflammasome itself. The NLR family includes a total of 17 members showing different structural features and ligand binding specificity [71], but an active inflammasome can also be assembled by more structurally distant PRRs like AIM2 or Pyrin [72]. The NLRP3 inflammasome is one of the best characterized and responds to a wide range of stimuli, including microbial infection, mitochondrial damage, and metabolic stress [73].

Autophagy is a common response to inflammasome activation that favors pathogen clearance [74], but it has also been involved in the negative regulation of inflammasome activity, mainly by promoting the degradation of the inflammasome itself or its individual components (Figure 2). For example, activated AIM2 and NLRP3 enable the G protein RalB to induce autophagosome formation which, in turn, sequester and degrade ubiquitinated ASC via selective autophagy mediated by p62 [75], thus providing a classical feedback regulatory loop that helps control the inflammatory response. Similarly, TLR signaling has been shown to induce PAI-2 expression which, in turn, stabilizes Beclin-1 to induce autophagy and autophagy-mediated NLRP3 degradation [76]. Autophagic degradation of NLRP3 is also regulated by tyrosine phosphorylation, as phosphorylated NLRP3 is preferentially targeted to autophagosomes over the non-phosphorylated form, providing an additional layer of regulation of the inflammatory response [77,78]. Intriguingly, mutations affecting the main NLRP3 phosphatase (PTPN22) are linked to increased risk of rheumatoid arthritis and systemic lupus erythematosus but protect against the intestinal inflammatory pathology Crohn’s disease [78], suggesting that upregulated NLRP3 inflammatory responses can have an unexpected beneficial effect in pathological inflammation. However, contradicting this view, impaired autophagy caused by the ATG16L1-T300A Crohn’s disease risk allele increases TLR/NLR-induced inflammatory responses accompanied by accumulation of p62 and enhanced levels of TRAF6/RIPK2 ubiquitination [79], suggesting in this case that NLR hyperactivity favors Crohn’s disease in the presence of the T300A allele. These seemingly conflicting scenarios indicate that the role of NLR signaling in inflammatory pathologies is not always straightforward and predictable. The current consensus is that NLRP3 signaling protects against a variety of infectious diseases by triggering moderate inflammation, but favors pathogenicity in a number of conditions that are accompanied by excessive inflammation, like Alzheimer’s or Parkinson’s diseases and Multiple Sclerosis, likely by contributing to the hyperinflammatory state (reviewed in [80]).

NOD family members assemble a RIPK2-based platform (called RIPosomes) that induces pro-inflammatory NF-kB signaling to fight bacterial infection. Recent studies show that the autophagy mediators IGRM and p62 can interact with NOD1/2 and RIPK2 to promote their degradation by selective autophagy, thus reducing inflammatory signaling [81,82]. Similarly, tripartite motif (TRIM) family proteins have been shown to act as selective autophagic receptors to degrade NLRP1, NLRP3 and pro-caspase-1 [83] as well as the AIM2 inflammasome, in this case with the intervention of p62 [84]. Interestingly, mutations in TRIM20 are risk factors for Familial Mediterranean Fever [83], a genetic autoinflammatory disease, thus again underscoring the importance of properly controlling inflammasome activity to prevent pathology. The variety of mechanisms mediating autophagic degradation of inflammasome components indicates the existence of a complex network of regulatory layers that keep inflammasome activity tightly controlled, consistent with the relevance that this regulation has for organismal homeostasis.

ATG16L1, a critical mediator of autophagy, has been shown to interact with NOD1/2 at the site of bacteria internalization to induce a defensive autophagic response that fights bacterial infection [85]. However, ATG16L1 also downregulates pro-inflammatory signaling by binding NOD2 and interfering with the poly-ubiquitination of RIPK2, thus preventing its recruitment to the NOD2 signaling platform [86]. These results point to a regulatory mechanism based on signaling interference rather than protein degradation. Interestingly, this activity of ATG16L1 is not recapitulated by other autophagy mediators (like ATG5 or ATG9) and can be uncoupled from the role of ATG16L1 in the autophagic pathway, indicating that it does not involve the canonical degradative function of autophagy. Such non-conventional role of ATG16L1 joins a growing collection of activities carried out by this mediator independently of its canonical function in the autophagic route. A similar interference-based mechanism has been described in microglial cells where, in response to LPS, cytosolic HMGB1 interacts with NOD2 to promote autophagy while simultaneously inhibiting proinflammatory signaling by disrupting NOD2-RIP interaction, revealing a pivotal node that helps balance inflammatory and autophagic signaling pathways [87].

Notably, as an exception in this chapter, ATG16L1 has also been shown to mediate a counterintuitive anti-inflammatory role of NOD1 in response to *Candida albicans* infection that proceeds through the selective autophagic degradation of the stress kinase ASK1 [88], pointing again to different context-dependent effects.

## 6. Role of Autophagy in the Control of RIG/MAVS Signaling

Cytosolic Retinoic acid-Inducible Gene-I (RIG-I)-like receptors (RLRs) are sensors of specific molecular features of viral RNAs and play important roles in the innate defense against viral infection [89]. They can also recognize endogenous RNA species of mitochondrial [90] or cytoplasmic [91] origin. This family of receptors includes RIG-I and Melanoma differentiation-associated protein 5 (MDA5), which, once stimulated, translocate to intracellular membranes (primarily mitochondria) to engage MAVS through their CARD domains, thus inducing the formation of large MAVS aggregates that are essential for function. Aggregated MAVS recruits TRAF family members for activation of IKK and TBK1 and downstream signaling to NF-KB and IRF-3 [92]. A complex network of protein modification and phosphorylation events tightly control RIG-I and MAVS activity and stability [93].

Autophagy has been involved in the regulation of RIG-I/MDA5-MAVS signaling, mostly by selective autophagic degradation of different members of the signaling system (Figure 2). For example, the ubiquitin ligases RNF144B and RNF167 ubiquitinate RIG-I and MDA5 upon their stimulation to induce their degradation via p62- [94,95] or CCDC50- [96] selective autophagy, helping in this way to control the inflammatory response. Other E3 ligases like MARCH8 or RNF34 induce K27 ubiquitination of MAVS to promote its selective autophagic elimination, in this case mediated by NDP52 [97,98]. Interestingly, in the first case, MARCH8 is recruited by a type-I IFN-responsive molecule induced by MAVS itself (Tetherin), thus establishing a classical feedback loop that controls MAVS activation during viral infection [97]. In the latter case, RNF34 promotes a transition from a K63 to K27 ubiquitination of activated MAVS that ensures its timely and progressive autophagic degradation to prevent excessive signaling, also promoting mitophagy during virus infection [98]. Other protein modifications besides ubiquitination induce the selective autophagic targeting of RIG-I. Thus, some viral infections cause RIG-I ISGylation to favor its binding to LRRC25 and degradation via p62-mediated autophagy [99,100]. Finally, an unconventional form of autophagy that does not appear to entail a degradative activity has been involved in the control of RIG-I signaling. In this case, the conjugate between ATG5 and ATG12 that plays a critical role in autophagosome formation directly interacts with the CARD domains of RIG-I and MAVS to prevent the assembly of early signaling complexes and inhibit downstream signaling [101,102]. This study points to a molecular interference mechanism by which ATG proteins control inflammatory responses, fundamentally different to their role in autophagy, and it is reminiscent to a similar function of ATG16L1 and HMGB1 in the regulation of NOD2 signaling (see above).

## 7. Role of Autophagy in the Control of STING Signaling

The cGAS/STING signaling system detects the abnormal presence of DNA in the cytoplasm, whether it is caused by viral infection, mitotic defects or mitochondrial permeabilization, and triggers an inflammatory response mediated by the activation of IRF3 and NF-kB transcription factors [103,104]. DNA fragments are initially converted by cGAS into cyclic GMP-AMP (cGAMP), which in turn activates STING in the endoplasmic reticulum. Activated STING traffics to Golgi-derived vesicles where it becomes phosphorylated by TBK1 for downstream signaling to IRF-3 and NF-kB activation.

Desensitization of STING signaling has been shown to involve the autophagic degradation of STING itself or some of its signaling components, at least in part through an autophagic response activated by downstream STING effectors [105,106] (Figure 2). Early studies showed that autophagy is a common response to STING activation to aid in the clearance of DNA and viral intermediates from the cytosol [107,108], an activity that, at least in some cases, appears to involve direct interaction between STING and the canonical autophagic effector WIPI2 [109]. Subsequent studies established that phosphorylation of p62 by TBK1 is a critical step that facilitates p62 recruitment to ubiquitinated STING, thus promoting the trafficking of active STING to autophagosomes [110]. Ubiquitination of STING after activation has been shown to be mediated by TRIM13 [111] or UBE2N [112]. The subsequent translocation of ESCRT to vesicles containing ubiquitinated STING is essential for STING degradation [112], a process that appears to involve a novel microautophagic mechanism where vesicles exhibiting lysosomal traits directly engulf the STING-positive compartments for degradation [113]. Notably, other studies have described an important role of Rab7-positive endolysosomal compartments in STING degradation without involvement of the autophagic machinery [114]. These results suggest that both autophagy-dependent and -independent mechanisms contribute to the control of STING signaling by lysosomal activity. Several studies have also identified the induction of an unconventional autophagic response driven by the proton channel activity of STING [115], which triggers endosomal alkalinization and a consequent vATPase- and ATG16L1-dependent Conjugation of Atg8 to Single Membranes (CASM) response that labels the same single-membrane vesicles where STING is located with LC3 [116,117]. This activity appears to restrain STING-induced cell death [117]. Importantly, a number of these reports show that interference with the STING degradation mechanisms at multiple levels causes elevation of proinflammatory signaling, offering a variety of possible therapeutic targets to boost innate immune signaling in those instances where it may be beneficial, like anti-cancer therapy [118]. However, possible therapeutic applications in this context are limited by the known pro-tumorigenic effects of STING signaling in the tumor microenvironment [119] and also the deleterious consequences that excessive and/or chronic inflammation has at the organismal level [120]. As an example of the latter, STING mediates the low-grade constitutive inflammation that accompanies aging, which is at least in part caused by the leakage of mtDNA from dysfunctional mitochondria [121]. Therefore, the control of STING signaling has complex implications in a wide variety of physiopathological processes.

## 8. Concluding Remarks and Future Prospects

While initially thought to be mainly a mechanism that degrades unspecific cytoplasmic material as an adaptation response to nutritional challenges, autophagy was quickly identified as a pathway that also eliminates selective components as a quality control routine and even degrades specific proteins to regulate a variety of signaling routes and molecular mechanisms. It is in the latter, selective modality that autophagy has a fundamental role in restraining inflammatory pathways to prevent excessive inflammatory reactions. The severity of the phenotypes observed at the organismal level when these autophagic control mechanisms are missing underscores their physiopathological relevance. However, a number of examples show that autophagy can also stimulate innate immune signaling or have unexpected effects in pathology in certain cases, indicating that its net effect in the control of inflammatory responses and overall pathophysiology is highly context-dependent (Table 1). More profound mechanistic knowledge about how autophagy controls the activity of innate immune pathways is expected improve the opportunities of effective therapeutic intervention in order to, first, control inflammatory processes by enhancing these autophagic regulatory mechanisms, and, second, boost innate immune signaling by inhibiting these mechanisms when it may be therapeutically beneficial, as in the case of stimulating anti-cancer immune responses. It is important to consider, however, that autophagy has pleiotropic roles in organismal homeostasis, so any intervention in the autophagic mechanisms directed to regulate innate immune signaling with a therapeutic purpose needs to be carefully evaluated to avoid unwanted side effects.

## Figures and Tables

**Table 1:** Pro-inflammatory and unexpected effects of autophagy or the autophagic machinery in innate immune signaling.

Physiological Role of Autophagy	Cellular Function of Autophagy	References
Pro-inflammatory	Mediates IL-1 secretion	[54,55]
Pro-inflammatory	Mediates ATP release during cell death	[49,50,51]
Pro-inflammatory	Mediates HMGB1 release during cell death	[47,48]
Pro-inflammatory	Promotes TLR ligand delivery to TLR containing endosomes	[66,67]
Pro-viral infection	Favors virus-induced PRR degradation	[35,36]
Protection against Crohn’s disease	Increased NLRP3 levels caused by reduced autophagic degradation in the presence of PTPN22 mutants	[78]
Anti-inflammatory	ATG16L1 mediates a paradoxical anti-inflammatory function of NOD1 in *C. albicans* infection	[88]

## Data Availability

No new data were created or analyzed in this study. Data sharing is not applicable to this article.

## Abbreviations

TLRs	Toll-like receptors
RIG	Retinoic acid-inducible gene
RLRs	Retinoic acid-inducible gene (RIG)-I-like receptors
NOD	Nucleotide-binding oligomerization domain
NLRs	Nucleotide-binding oligomerization domain-like receptors
STING	Stimulator of interferon genes
PAMPs	Pathogen-associated molecular patterns
PRRs	Pattern recognition receptors
DAMPs	Danger-associated molecular patterns
cGAS	Cyclic GMP–AMP synthase
ATGs	Autophagy-related proteins
mTOR	Mechanistic target of rapamycin
LANDO	LC3-associated endocytosis
LAP	LC3-associated phagocytosis
MAVS	Mitochondrial antiviral signaling protein
LIR	LC3-interacting region
SQSTM	Sequestosome
NBR	Neighbor of BRCA1
NDP	Nuclear dot protein
OPTN	Optineurin
LPS	Lipopolysaccharide
MDP	Muramyl dipeptide
mtDNA	Mitochondrial DNA
ROS	Reactive oxygen species
HMGB	High mobility group box
MDVs	Mitochondria derived vesicles
MVBs	Multivesicular bodies
TRIM	Tripartite motif protein
MDA	Melanoma differentiation-associated protein
cGAMP	Cyclic guanosine monophosphate–adenosine monophosphate
CASM	Conjugation of ATG8 to single membranes
